# The Probilin Treatment of Cholelithiasis

**Published:** 1906-09

**Authors:** 


### ABSTRACTS.

### The Probilin Treatment of Cholelithiasis

IN May, 1904, Dr. W. Bauermeister published in the Therap. Monatshefte his results from the treatment of gallstone disease with probilin, a pill composed of acid sodium oleate, salicylic acid, menthol and phenolphthalein. Salicylic acid is an acknowledged cholagogue and biliary disinfectant; acid sodium oleate is also a recognised cholagogue; menthol is an analeptic and carminative; and phenolphthalein stimulates intestinal activity.

Since then, four years ago, he has seen probilin give brilliant results in the very severest attacks of cholelithiasis. But he bases his conclusions of its efficiency only upon cases that he treated two years ago or longer. To 80 such patients, mostly dating from 1902 and 1903, he sent letters of inquiry, so as to get their own opinions; for gallstone disease is an affection concerning which the sufferer can judge with great exactitude whether his malady has persisted. The following questions were put: Have you been cured? If so, by what? Or, do you feel much or only a little improved? Or, have you noted no effect whatever?

To these 80 inquiries 42 answers were received. Doubters may claim that the missing replies represent polite disapproval; but such is not his opinion. The few disapprovals came in first; for the public nowadays is much quicker to blame than to praise. Besides, he knows positively that quite a number who failed to answer are in good health and entirely free from trouble.

Four patients disapproved of probilin. One was a gentleman who suffered severely from cholelithiasis for about five years and who, after trying everything, was operated on 2½ years before he tried probilin. Soon after the operation his old troubles reappeared, and he claims that probilin had no effect. His present freedom from disease he ascribes to some household remedy, whose nature he would not reveal. The second patient did see some results from probilin, but they did not endure. It was only after using tea and oil that she passed 21 stones, and since then she had no pain. I am doubtful as to the passage of concretions, since the examination of the so-called calculi passed under oil treatment shows that they are merely saponified masses found in the intestines, not stones. The third patient also says he got relief after oil treatment. The fourth patient states that even after the probilin treatment attacks occurred at intervals of 6 to 8 weeks. She had other treatment after it, including the Neuenahr and Carlsbad, without avail.

Seventeen patients replied that they were improved or greatly improved and consider their present condition fairly good or very good. Most of them have been through varied methods of treatment, so that we must lay special stress on the improvement they note. Experience has taught them to be cautious enough to speak only of improvement, even if they have had no attack for a year.

Twenty-one patients state explicitly that they are cured; that is, that since the first probilin course,—in many cases taken three years before,—there has been no sign of the disease. Some of these cases were light, but many of them were severe, and for not a few the operating table was already waiting.

Thus 4 patients were not influenced, 17 were improved, and 21 were entirely cured. Similar results have been reported to me by other physicians. We see stones voided under probilin with a frequency entirely unknown under other medication; colic with the passages is usually light and may be absent, though sometimes it is marked.—*Therap. Monatshefte,* March, 1906.

